# Clinical relevance of patient-reported outcome measures in the surgical management of focal chondral defects of the knee: a systematic review

**DOI:** 10.1186/s10195-025-00897-0

**Published:** 2026-01-16

**Authors:** Filippo Migliorini, Nicola Maffulli, Michael Kurt Memminger, Ulf Krister Hofmann

**Affiliations:** 1 Department of Trauma and Reconstructive Surgery, Martin-Luther University Halle-Wittenberg, University Hospital of Halle, 06097 Halle (Saale), Germany; 2 Department of Orthopedics and Trauma Surgery, Academic Hospital of Bolzano (SABES-ASDAA), 39100 Bolzano, Italy; 3 Department of Life Sciences, Health, and Health Professions, Link Campus University, 00165 Rome, Italy; 4 Department of Trauma and Orthopaedic Surgery, University La Sapienza, University Hospital Sant’ Andrea, 00185 Rome, Italy; 5 School of Pharmacy and Bioengineering, Faculty of Medicine, Keele University, Stoke on Trent, ST4 7QB UK; 6 Centre for Sports and Exercise Medicine, Barts and the London School of Medicine and Dentistry, Mile End Hospital, Queen Mary University of London, London, E1 4DG UK; 7 Department of Orthopaedic, Trauma, and Reconstructive Surgery, RWTH University Medical Centre, 52074 Aachen, Germany; 8 Department of Orthopaedic, Trauma, and Reconstructive Surgery, Division of Arthroplasty and Tumour Surgery, RWTH University Medical Centre, Pauwelsstraße 30, 52074 Aachen, Germany

**Keywords:** Cartilage, MCID, PASS, SCB, MDC, CID, PROMs

## Abstract

**Introduction:**

To evaluate clinical outcome following surgical management of focal chondral defects in the knee, patient-reported outcome measures (PROMs) are used. To give these measures meaning, parameters such as the minimal clinically important difference (MCID), patient-acceptable symptom state (PASS), minimally detectable change (MDC), clinically important difference (CID) and substantial clinical benefit (SCB) have been introduced. This systematic review investigated the MCID, SCB, CID, PASS and MDC of the most commonly used PROMs for assessing patients following surgical repair of focal chondral defects of the knee.

**Methods:**

This systematic review was conducted in accordance with the 2020 Preferred Reporting Items for Systematic Reviews and Meta-Analyses (PRISMA) guidelines and the recommendations of the Cochrane Handbook for Systematic Reviews of Interventions. All clinical studies investigating tools to assess the clinical relevance of PROMs in the surgical repair of focal chondral defects of the knee were reviewed. In April 2025, the following databases were accessed: PubMed, Web of Science and Embase. The PROMs of interest included: the International Knee Documentation Committee (IKDC) questionnaire, the Knee Injury and Osteoarthritis Outcome Score (KOOS) and its related subscales activities of daily living (ADL), pain, quality of life (QoL), sports and recreational activities, and symptoms, the Western Ontario and McMaster Universities Osteoarthritis (WOMAC) score, the Tegner Lysholm knee scoring scale, the Short Form-12 (SF-12) and its related mental and physical component subscales, the Short Form-36 (SF-36) and the Cincinnati Knee Rating System (CKRS). The Risk of Bias in Nonrandomised Studies of Interventions (ROBINS-I) indicated a low to moderate risk of bias.

**Results:**

The systematic literature search yielded 524 articles. Only data from four studies (involving 421 patients) were included. All of these were non-randomised controlled trials (RCTs) employing a retrospective study design. Most reported thresholds for a significant change across the questionnaires ranged from 20 to 30 points on a 100-point scale, whereas PASS values ranged from 62 points in the IKDC to 87 points in the KOOS ADL.

**Conclusions:**

Despite a comprehensive search strategy, only four studies met the inclusion criteria, underscoring that the parameters analysed remain overlooked in the scientific literature. Reported results for MCID, CID and MDC following cartilage repair are relatively consistent in magnitude, ranging from 10 to 20. Differences reported in the literature that fall below this range should be regarded as no improvement. For SCB and PASS, values were even higher, spanning from 20 to 30 and from 62 to 87 points in IKDC and KOOS ADL, respectively. Given the high standard of modern medical care, further development and validation of condition-specific PROMs should be considered to facilitate future clinical evaluations using PROMs.

*Level of evidence*: Level III, systematic review.

**Supplementary Information:**

The online version contains supplementary material available at 10.1186/s10195-025-00897-0.

## Introduction

Over the past decades, several surgical techniques have been established to manage cartilage defects [1, 2], including debridement and shaving, microfracture and subchondral drilling, osteochondral autograft transfer (OAT) and osteochondral allograft transfer (OCA), autologous chondrocyte implantation (ACI) and treatment with particulated cartilage as allo- or autograft [3–5]. Each of these techniques has its own advantages and drawbacks [6–8]. In addition, clinical outcomes vary with defect size, defect location and chronicity [9–12]. The interaction between patient-related factors such as age, body mass index and activity level has well been established [13, 14]. In the context of cartilage repair, the goal should always be to fully restore the patient’s function and to provide substantial symptom relief [15, 16]. Given the abundance of techniques and the diversity of cartilage defects, it is evident that treatment choice is always an individual decision. Data from the literature, consensus meeting guidelines, personal experience and qualifications inform this decision-making process. The exact level of evidence for these recommendations, however, is not always clear. One reason is that reporting study results often permits considerable misinterpretation. One of the significant developments in medicine over the past few decades has been the establishment of a clear philosophy for evidence-based medicine, which is the conscientious, explicit and judicious use of current best evidence in making decisions about the care of individual patients [17]. A breakthrough in reporting medical data was the introduction of a *p*-value cut-off of 0.05 [18]. Having found widespread use in the medical literature, results have been progressively categorised as statistically significant or insignificant [19]. Whilst this concept of signalling the probability of error concerning a null hypothesis can help to discriminate relevant from irrelevant data, it bears a few risks that might be underestimated in a clinical setting and which are mostly related to sample size: First, clinically relevant differences might be overlooked owing to a type II error (false negative result). The risk of a type II error is especially high in clinical studies, as they are often underpowered [20, 21]. Of note, although less intuitively, low power also reduces the probability that a statistically significant result reflects a true effect [20]. Second, even the smallest conceivable difference can become statistically significant when the sample size is simply large enough. Notably, such a small difference may not be clinically relevant, as statistical significance does not imply clinical importance. Believing that a statistically significant result implies a clinically meaningful finding can lead to an erroneous interpretation of the results [22].

For these reasons, new concepts have been introduced to quantify the magnitude of therapeutic effects in a clinically relevant context [23]. In many cases, evaluating the therapeutic success of a treatment requires the systematic, evidence-based analysis of patient-reported outcome measures (PROMs). These PROMs typically comprise a set of questions addressing features of the treated organ or its implications for daily living. As such, outcomes are interval-scaled data with no absolute definitions for interpreting individual values. To correctly interpret treatment effects, such values would, however, be essential. For this reason, Jaeschke et al. proposed the term minimal clinically important difference (MCID) (later also termed minimal important difference, MID) [24]. It is considered the smallest change in a treatment outcome that an individual patient would identify as important and which would indicate a change in the patient’s management [25]. Over the years, other clinically oriented concepts have been introduced. While the MCID, from a therapeutic perspective, defines the minimum acceptable change, the magnitude of health-related quality-of-life improvement a patient still recognises as a substantial benefit was termed the substantial clinical benefit (SCB) [26]. The clinically important difference (CID) is the difference in outcome scores between two groups that can be clinically relevant [27]. The patient-acceptable symptom state (PASS) is the value on a PROM scale beyond which patients consider themselves well or in a satisfactory state [28]. Finally, the minimal detectable change (MDC) is used to determine the threshold for whether a pre–post change is true from a measurement perspective [29, 30]. It is the error associated with two scores, or change scores. It is calculated as the standard deviation (SD) (from the study on test–retest reliability) x

$\sqrt{(1-\text{intraclass correlation coefficient})} \times \sqrt{2}$ [30]. Using these parameters, a more accurate, patient-oriented description of the observed success rates in therapeutic approaches can be achieved. To interpret scientific findings correctly, results should be placed in the context of these parameters. This systematic review evaluated the currently available data on MCID, SCB, CID, PASS and MDC for the most commonly used PROMs to assess patient outcomes following surgical management of focal chondral defects of the knee. The present study aimed to provide an overview of the existing literature on these parameters in the field and to highlight the still-relevant limitations of the reported data.

## Methods

### Eligibility criteria

All clinical studies investigating tools to assess the clinical relevance of PROMs in the surgical management of focal chondral defects of the knee were accessed. Only surgical procedures performed on the knee were considered. Only studies which evaluated the MCID, SCB, CID, PASS or MDC were eligible. According to the authors’ language capabilities, articles in English, German, Italian, French and Spanish were eligible. Only studies with levels I–IV of evidence, according to the Oxford Centre of Evidence-Based Medicine [31], were considered. Reviews, opinions, letters and editorials were not considered. Missing quantitative data for outcomes of interest warranted exclusion of the study.

### Search strategy

This study was conducted according to the Preferred Reporting Items for Systematic Reviews and Meta-Analyses: the 2020 PRISMA statement [32]. The PICOD algorithm was preliminarily established:

- P (problem): focal chondral defects of the knee
- I (intervention): surgical repair (arthroscopy or arthrotomy) of focal chondral defects
- C (comparison): clinical efficacy of surgery
- O (outcomes): MCID, SCB, CID, PASS and MDC
- D (design): clinical study

In April 2025, the following databases were accessed: PubMed, Web of Science and Embase. No time constraint was set for the search. The Medical Subject Headings (MeSH) used for the database search are reported in the Appendix. No additional filters were applied to the database search, and no additional time constraints were imposed.

### Selection and data collection

Two authors (F.C. and M.M.) independently performed the database search. All resulting titles were screened by hand, and if suitable, the abstract was accessed. The full text of the abstracts which matched the topic was accessed. If the full text was unavailable, the article was not considered for inclusion. A cross-reference of the full-text article bibliographies was also performed for inclusion. Disagreements were resolved by the authors. In case of further disagreements, a third senior author (N.M.) made the final decision.

### Data items

Two authors (F.C. and M.M.) independently performed the data extraction. The following baseline data were extracted: author, year of publication, journal, country, number of patients, type of PROMs investigated and type of analysis performed. Data on the MCID, SCB, CID, PASS and MDC were collected. The PROMs of interest were the International Knee Documentation Committee (IKDC) questionnaire [33], the Knee Injury and Osteoarthritis Outcome Score (KOOS) and its related subscales activities of daily living (ADL), pain, quality of life (QoL), sports and recreational activities, and symptoms [34], the Western Ontario and McMaster Universities Osteoarthritis (WOMAC) score [35, 36], the Tegner Lysholm knee scoring scale [37], the Short Form-12 (SF-12) and its related mental and physical component subscales [38], the Short Form-36 (SF-36) [39–42] and the Cincinnati Knee Rating System (CKRS) [43]. Data were extracted in Microsoft Office Excel version 16.72 (Microsoft Corporation, Redmond, USA).

### Methodological quality assessment and quality of the recommendations

The risk of bias was assessed in accordance with the Cochrane Handbook for Systematic Reviews of Interventions [44]. Two reviewers (F.C. and M.M.) independently evaluated the risk of bias in the extracted studies. Disagreements were solved by a third senior author (N.M.). Given the lack of randomised controlled trials (RCTs), the Risk of Bias in Nonrandomised Studies of Interventions (ROBINS-I) tool was used [45]: Seven domains of potential bias in non-RCTs are assessed. Possible confounding factors and the nature of patient selection before the start of the comparative intervention are assessed within two domains. A further domain is used to evaluate classification bias during the intervention. The final four domains assess methodological quality after the start of the intervention, evaluating biases arising from deviations from the intended initial interventions, missing data, erroneous measurement of outcomes or bias in the selection of the reported outcomes. The ROBINS-I figure was developed using the RobVis software (Risk-of-bias VISualization; RiskofBias.info, Bristol, UK) [46].

### Synthesis methods

The main author (F.M.) performed the statistical analyses following the recommendations of the Cochrane Handbook for Systematic Reviews of Interventions [47]. The arithmetic mean was calculated using IBM SPSS Statistics version 25 (International Business Machines Corporation, Armonk, USA) for descriptive statistics.

## Results

### Study selection

**Fig. 1:**
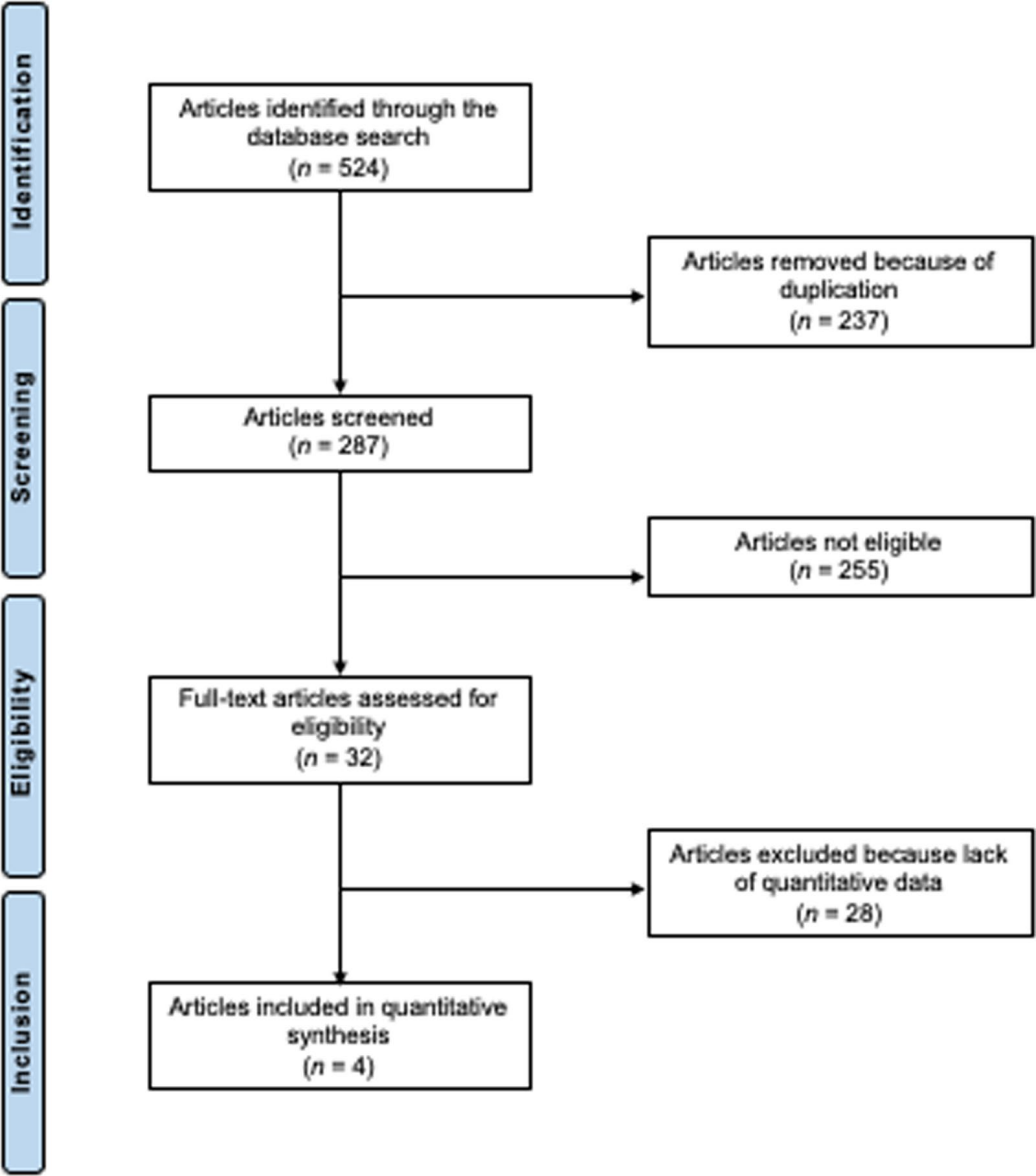
PRISMA flow chart of the literature search

The systematic literature search resulted in 524 articles. A total of 237 were identified as duplicates and therefore excluded. A further 255 investigations were discarded as they did not meet the defined inclusion criteria. In particular, the reasons for ineligibility included not matching the topic (*N* = 155), inadequate type of article (review, opinion, letter or editorial, *N* = 3), low level of evidence (*N* = 7), surgery procedures performed not in the knee (*N* = 33), missing implementation of at least one tool to determine the clinical relevance of PROMs (MCID, SCB, CID, PASS or MDC) (*N* = 21), not reporting data from at least one PROM of interest (IKDC, KOOS, WOMAC, Tegner Lysholm score, SF-12, SF-36 or CKRS) (*N* = 29) and language limitations (*N* = 7). After full-text evaluation, an additional 28 investigations were excluded because they did not offer quantitative data on the outcomes of interest. Finally, four studies were included. All were non-RCTs with retrospective study designs. The results of the literature search are shown in Fig. 1.

### Methodological quality assessment

**Fig. 2:**
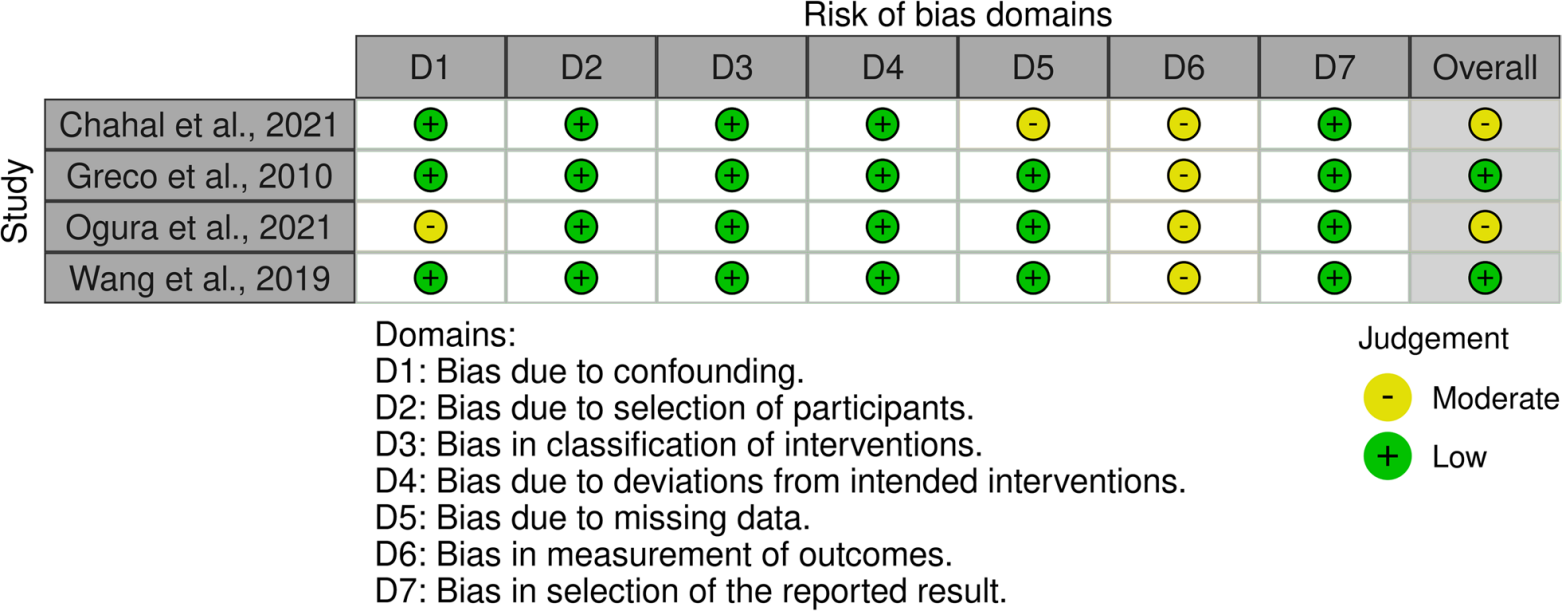
The ROBINS-I of non-RCTs

The ROBINS-I was used to assess risk of bias across all articles included in the present review. No study was rated as having an overall serious or critical risk of bias. Most authors reported high-quality allocation concealment, resulting in comparable study groups at baseline, and intervention assignment was protected from prognostic variables, leading to a low risk of bias from confounding and participant selection. The risk of bias in the classification of intervention yielded uniformly low values, as neither non-differential nor differential misclassification was identified. Some studies lacked reported outcome data. Therefore, they were rated as having a moderate risk of bias due to missing data. The lack of assessor blinding in all studies reviewed resulted in a moderate risk of bias in measuring outcomes in every investigation asset. No concerns were raised about the risk of bias from selecting the reported results for any of the studies. Given the mainly high methodological quality of the included studies, the overall risk of bias was low to moderate (Fig. 2).

### Study characteristics and results of individual studies

Data from 421 patients were collected. Three studies performed arthroscopy [48–50], and one author reported data from open surgery [51]. Two studies performed osteochondral allograft transplantation [50, 51], one study performed debridement, microfracture, osteochondral transfer, ACI and particulated cartilage [48], and another study performed debridement, drilling, ACI, microfracture and cell therapy [49]. All studies were retrospective cohort studies. Three studies evaluated the MCID [49–51], two the SCB [50, 51], one the CID [48], one the PASS [48] and one the MDC [49]. The PROMs evaluated were the IKDC [48–51], the KOOS [48, 50, 51], the Lysholm [48], the WOMAC [49], the CKRS [49] and the SF-36 [49] and SF-12 [51]. All studies were conducted in the USA between 2010 and 2021. The general characteristics and demographics of the included studies are presented in Table 1.

**Table 1:** General characteristics, sample size and applied PROMs of the included studies

Authors	Year	Country	Design	Journal name	Procedure	Exposure	Type of analyses	PROMs	Patients (*n*)
Chahal et al. [48]	2021	USA	Retrospective	*Am J Sports Med*	Debridement, microfracture, osteochondral transfer, ACI, particulated cartilage	Arthroscopy	CID/PASS	KOOS, IKDC, Lysholm	113
Greco et al. [49]	2010	USA	Retrospective	*Am J Sports Med*	Debridement, ACI, microfracture, cell therapy	Arthroscopy	MCID/MDC	IKDC, WOMAC, CKRS, SF-36,	49
Ogura et al. [51]	2021	USA	Retrospective	*Cartilage*	OCA	Arthrotomy	MCID/SCB	KOOS, IKDC, SF-12	86
Wang et al. [50]	2019	USA	Retrospective	*Am J Sports Med*	OCA	Arthroscopy	MCID/SCB	IKDC, KOOS	173

ACI, autologous chondrocyte implantation; CID, clinically important difference; CKRS, Cincinnati Knee Rating System; IKDC, International Knee Document Committee; KOOS, Knee Injury and Osteoarthritis Outcome Score; MCID, minimal clinically important difference; MDC, minimal detectable change; OCA, osteochondral allograft transplantation; PASS, patient acceptable symptom state; PROMs, patient-reported outcome measures; SCB, substantial clinical benefit; SF, Short Form; WOMAC, Western Ontario and McMaster Universities Osteoarthritis

### Results syntheses

An overview of the results of MCID, SCB, CID, PASS and MDC with respect to the IKDC, KOOS, Tegner Lysholm score, SF-36 and WOMAC score is reported in Table 2. The International Knee Documentation Committee (IKDC) score showed relatively consistent estimates with an MCID of 17.4, SCB of 28.5, CID of 9.2, PASS of 62.1 and MDC of 18.3. Among the KOOS subscales, MCID values ranged from 10.0 (ADL) to 19.2 (Symptoms and Sports/Rec), SCB values from 19.8 to 28.9 and CID values from 8.6 to 21.8. PASS values for KOOS subscales ranged widely, from 54.3 (Sports/Rec) to 86.8 (ADL), suggesting domain-specific patient expectations. The Tegner Lysholm score showed higher thresholds, with an MCID of 19.2, SCB of 22.4, CID of 14.6 and PASS of 66.4. MDC values were also available for the WOMAC and SF-36 scores, although they were reported in only one study and with a smaller sample size (*n* = 49). Notably, WOMAC stiffness had the highest MDC (30.6), followed by WOMAC pain (14.4), physical function (15.0) and overall (15.3). For the SF-36, MDC values varied across subdomains, with the highest reported for the role-emotional domain (36.3) and the lowest for the physical component summary (6.6). Data for the SF-12 were not reported in any included study. The CKRS demonstrated an MCID of 26.0 and an MDC of 22.8, although these estimates are based on limited data. These findings highlight a relatively homogenous range for MCID, CID and MDC, typically between 10 and 20 points across most scales, whilst SCB thresholds were generally higher, ranging from 20 to 30. PASS values, in contrast, varied more substantially across functional domains.

**Table 2:** Main results

PROMs	Patients (*n*)	MCID	SCB	CID	PASS	MDC
IKDC (0–100)	384	17.4	28.5	9.2	62.1	18.3
KOOS ADL (0–100)	286	10.0	21.0	8.8	86.8	
KOOS Pain (0–100)	485	13.4	24.4	8.6	79.5	
KOOS QOL (0–100)	598	13.4	27.8	13.7	64.8	
KOOS Sports/Rec (0–100)	711	19.2	28.9	21.8	54.3	
KOOS Symptoms (0–100)	824	19.2	19.8	16.3	62.9	
WOMAC (pain) (0–20)	49	7.5				14.4
WOMAC (physical function) (0–68)	49	5.9				15.0
WOMAC (stiffness) (0–8)	49	18.8				30.6
WOMAC (overall) (0–96)	49	11.5				15.3
Tegner Lysholm (0–100)	937	19.2	22.4	14.6	66.4	
SF-36 (bodily pain) (0–100)	49					26.6
SF-36 (general health) (0–100)	49					21.4
SF-36 (MCS) (0–100)	49	3				10.0
SF-36 (mental health) (0–100)	49					20.8
SF-36 (PCS) (0–100)	49	4.6				6.6
SF-36 (physical functioning) (0–100)	49	17.5				11.0
SF-36 (role emotional) (0–100)	49					36.3
SF-36 (role physical) (0–100)	49	12.5				35.3
SF-36 (social functioning) (0–100)	49					29.5
SF-36 (vitality) (0–100)	49	2.6				33.1
CKRS (6–100)	49	26.0				22.8

ADL, activities of daily living; CID, clinically important difference; CKRS, Cincinnati Knee Rating System; IKDC, International Knee Documentation Committee questionnaire; KOOS, Knee Injury and Osteoarthritis Outcome Score; MCID, minimal clinically important difference; MCS, mental component summary; MDC, minimally detectable change; PASS, patient-acceptable symptom state; PCS, physical component subscale; PROMs, patient-reported outcome measures; QoL, quality of life; Rec, recreational activities; SCB, substantial clinical benefit; SF, Short Form; WOMAC, Western Ontario and McMaster Universities Osteoarthritis. In brackets: the range of points the scale offers

## Discussion

In the present study, MCID, SCB, CID, PASS and MDC were systematically reviewed across established PROMs used in the surgical management of focal chondral defects of the knee. In total, four studies were included, all non-RCTs from the USA with a retrospective design. Most reported values for a relevant change across the questionnaires ranged from 20 to 30 points on a 100-point scale. Values for PASS ranged from 62 points in the IKDC to 87 points in the KOOS ADL. In a longitudinal cohort study to determine the responsiveness of the IKDC, the MCID was calculated as 11.5 and the MDC as 12.8 [52]. These values are slightly lower than those reported in the analysed literature in the present work, at 17.4 for the MCID and 18.3 for the MDC. The relationship between the two values, however, is similar. In a systematic review, Collins et al. calculated a distribution-based MDC for the KOOS subscales, ranging from 14.3 to 19.6 for younger individuals and more than 20 for older individuals after knee injury [53]. In a study on patients with knee osteoarthritis, Harris et al. reported an anchor-based MCID of 12 and an MDC of 16 [54]. Although we did not find any data on MDC in cartilage repair procedures, the MCID data are consistent with those reported in studies of other knee conditions. In a recent retrospective analysis of patients with recurrent patellar instability treated with medial patellofemoral ligament repair and tibial tubercle transfer, Qiao et al. [55] reported a distribution-based MCID for the Tegner Lysholm scale of 11.1 and 9.9 for the IKDC. The anchor-based SCB was 12.5 for the Tegner Lysholm scale and 14.5 for the IKDC.

A striking feature of these reported results is the relatively homogeneous magnitude of MCID, CID and MDC. Values reported across all tools range from 10 to 20. This also applies to knee conditions other than cartilage defects and repair strategies (see above). This is noteworthy in several ways. First of all, reported differences in the literature smaller than this range were considered to be of no improvement. Second, the magnitude of MCID and CID should be considered when designing future studies and calculating sample sizes. When trying to show an SCB, the values reported appear even higher, ranging from 20 to 30. Applying such a demanding level of improvement in PROMs will make it almost impossible to test the superiority of one treatment in a study design if baseline values are not low. More detailed, condition-specific questionnaires may be required in this context to enable substantial improvements at a higher level, as do currently established PROMs such as the KOOS and the IKDC. For example, the content validity of the KOOS in patients with ACL injury has been questioned [56]. Also, regularly updating these questionnaires to changing language and social needs might be advisable [57]. Recommendations on optimising face and content validity on a condition-specific PROM have already been developed [58]. Given the high quality of modern medical care, further development and validation of condition-specific PROMs can be recommended [59].

One dimension that appears less dependent on the mode of reporting is pain. Georgopoulos et al. [60] reported a comparable level of pain, as assessed using the KOOS, the WOMAC, the numeric rating scale and the visual analogue scale. In contrast, the PASS appeared to differ substantially depending on the underlying condition that led to the ascribed pain levels. In a scale adjusted for all four PROMS from 0 (no pain) to 100 (worst pain), PASS scores were lowest in ligament tears (12, 95% CI 11–13). Comparable PASS pain levels were stated for knee osteoarthritis (31, 95% CI 26–36) and meniscal tears (27, 95% CI 20–35), which were more than two-fold higher than for ligament rupture. In the present systematic review of cartilage repair procedures, the PASS value for pain, when transformed to the scale used by Georgopoulos et al. [60], was 20.5 in the KOOS, indicating a level between ligament rupture and osteoarthritis and meniscal tear. The reasons for this difference remain speculative. Certainly, a ligament tear is a sudden injury, with clinical symptoms that arise immediately. In contrast, osteoarthritis and degenerative meniscal tears develop gradually over time, with intermittent phases of improvement and deterioration. Between these two extremes lies the development of symptoms associated with cartilage defects. Thus, the PASS may be strongly influenced by patients’ cognitive factors, such as expectations, coping strategies and habits. Notably, the PASS for activities of daily living was much higher (86.8) than that for putatively optional dimensions such as sports and recreational activities (54.3). One aspect that needs to be stressed, however, is that there is as yet no unanimous definition of how such values are actually to be calculated. The major distinction in the calculation of values such as the MCID is made between anchor-based approaches and distribution-based approaches [61]. Whereas purely distribution-based threshold definitions might actually not reflect the patient’s perception of a condition, internal anchors provide a real-world basis for interpreting changes in clinical outcomes, calculating parameters such as the MCID more meaningfully and applying to clinical practice. They help to avoid purely statistical thresholds and ensure that the findings align with patient perceptions and clinical relevance. Owing to the purely subjective nature of these internal anchors and an expected inter-individual variability, it has been suggested that the order of selection of anchors should generally be: objective anchors > anchors with established MCID in subjective anchors (specific scale > generic scale) > ranked anchors in subjective anchors, whereby all anchors should be evaluated by a correlation test [62]. Subjective anchors, however, might not always be available in retrospective studies. In these instances, distribution-based anchors might be an option.

Despite a thorough literature search, only four studies could be included that reported quantitative data on MCID, SCB, CID, PASS and MDC in the context of knee cartilage repair. Notably, all articles were from the USA, underscoring that this concept may not yet have reached global attention. The number of patients included in the data collection for the WOMAC, SF-36 and CKRS is thus low, with only *n* = 49. The fact that all data were collected in retrospective study designs further limits the generalisability of the observed data, since subjective internal anchor data may not always have been available with sufficient discriminative power, as might be the case in a prospective study. The scarcity and heterogeneity of the data also led to seemingly implausible results, as values reported for the CID were, for example, mostly smaller than those for the MCID and the MDC. On the basis of our data, we can confirm that threshold values may need to be condition- and procedure-specific. We therefore strongly advocate the regular use and reporting of such parameters in future studies to improve comparability across studies and treatment outcomes. Given the high degree of differentiation and specialisation of modern medical care, further development and validation of condition-specific PROMs should be considered to facilitate clinical advancements evaluated by PROMs.

Studies investigating the surgical management of chondral defects of the knee are abundant in the current literature, yet very few have focused on the psychometric foundations required to determine whether a statistically significant improvement is also clinically meaningful. This gap has led to a persistent ambiguity in translating quantitative results into patient-centred outcomes. The present review provides a valuable reference for researchers by summarising the thresholds that define clinically relevant changes in PROMs, thereby facilitating a more informed interpretation of future findings. Moreover, the study highlights the need for a unified methodological approach, encouraging the orthopaedic community to integrate clinical relevance as a fundamental dimension of evidence assessment. By advocating further research to refine these psychometric parameters, this review emphasises that the ultimate goal of cartilage repair should not merely be statistical improvement but tangible functional recovery as perceived by the patient.

## Conclusions

Despite a broad search strategy, only four studies met the inclusion criteria, underscoring that the analysed parameters remain under-represented in the scientific literature. Reported results for MCID, CID and MDC after cartilage repair are relatively homogeneous in magnitude, ranging from 10 to 20, suggesting that differences in the literature smaller than this range should be considered as no improvement. For SCB and PASS, values were even higher, spanning from 20 to 30 and from 62 to 87 points in IKDC and KOOS ADL, respectively. Given the high standard of modern medical care, further development and validation of condition-specific PROMs should be considered to facilitate future clinical evaluations using PROMs.

## Supplementary Information

Supplementary Material 1.

Supplementary Material 2.

## Data Availability

The datasets generated during and/or analysed during the current study are available throughout the manuscript.
