# Supplementary material for: Clinical relevance of patient-reported outcome measures in the surgical management of focal chondral defects of the knee: a systematic review
Source: J Orthop Traumatol. 2026 Jan 16;27:3. doi: 10.1186/s10195-025-00897-0 (PMC12847531; doi:10.1186/s10195-025-00897-0)
Supplement: Supplementary file 1 — Supplementary Material 1. [file 10195_2025_897_MOESM1_ESM.docx]

**Systematic literature search for:**

**Minimal clinically important difference (MCID), patient-acceptable symptom state (PASS), minimally detectable change (MDC), clinically important difference (CID), and substantial clinical benefit (SCB) in patients following surgical repair of focal chondral defects of the knee: a systematic review**

**Concept 1: Surgical repair of focal chondral defects of the knee**

**Keywords:**

**knee**

focal chondral defects

chondral defect

cartilage repair

cartilage restoration

**Mesh:**

"Knee Joint"[Mesh]

"Knee Joint/surgery"[Mesh]

"Cartilage Diseases/surgery"[Mesh]

"Cartilage, Articular/surgery"[Mesh]

"Arthroplasty, Subchondral"[Mesh]

**Concept 3: Interest in Outcome**

**Keywords**

**clinical efficacy of surgery**

minimal clinically important difference (MCID)

patient-acceptable symptom state (PASS)

minimally detectable change (MDC)

clinically important difference (CID)

substantial clinical benefit (SCB)

**Mesh:**

"Treatment Outcome"[Mesh]

"Minimal Clinically Important Difference"[Mesh]

**PROMS**

Knee Injury and Osteoarthritis Outcome Score (KOOS)

KOOS activity of day living (ADL)

KOOS pain

KOOS quality of life (QoL)

KOOS sport/recreational

KOOS symptoms

Lysholm knee scoring scale

Short Form 12 (SF-12)

Short Form 36 (SF-36)

Cincinnati Knee Rating System (CKRS)

Western Ontario and McMaster Universities Osteoarthritis (WOMAC)

**Mesh:**

"Treatment Outcome"[Mesh]

"Patient Outcome Assessment"[Mesh]

"Patient Reported Outcome Measures"[Mesh]

"Visual Analog Scale"[Mesh]

"Knee Injuries"[Mesh]

"Quality of Life"[Mesh]

"Lysholm Knee Score"[Mesh]

**Concept 1: Surgical repair of focal chondral defects of the knee**

"Knee Joint"[Mesh] OR knee

**AND**

"Knee Joint/surgery"[Mesh] OR "Cartilage Diseases/surgery"[Mesh] OR "Cartilage, Articular/surgery"[Mesh] OR "Arthroplasty, Subchondral"[Mesh] OR focal chondral defects OR chondral defect OR cartilage repair OR cartilage restoration

**AND**

**Concept 3: Interest in Outcome**

"Treatment Outcome"[Mesh] OR "Minimal Clinically Important Difference"[Mesh] OR clinical efficacy of surgery OR minimal clinically important difference OR MCID OR patient-acceptable symptom state OR PASS OR minimally detectable change OR MDC OR clinically important difference OR CID OR substantial clinical benefit OR SCB

**AND**

"Patient Outcome Assessment"[Mesh] OR "Patient Reported Outcome Measures"[Mesh] OR "Visual Analog Scale"[Mesh] OR "Knee Injuries"[Mesh] OR "Quality of Life"[Mesh] OR "Lysholm Knee Score"[Mesh] OR PROMS OR Knee Injury and Osteoarthritis Outcome Score OR KOOS OR KOOS activity of day living OR KOOS ADL OR KOOS pain OR KOOS quality of life OR KOOS QoL OR Lysholm knee scoring scale OR Short Form 12 OR SF-12 OR Short Form 36 OR SF-12 OR Cincinnati Knee Rating System OR CKRS OR Western Ontario and McMaster Universities Osteoarthritis OR WOMAC

**Summary of the Search**

((("Knee Joint"[Mesh] OR knee) AND ("Knee Joint/surgery"[Mesh] OR "Cartilage Diseases/surgery"[Mesh] OR "Cartilage, Articular/surgery"[Mesh] OR "Arthroplasty, Subchondral"[Mesh] OR focal chondral defects OR chondral defect OR cartilage repair OR cartilage restoration)) AND ("Treatment Outcome"[Mesh] OR "Minimal Clinically Important Difference"[Mesh] OR clinical efficacy of surgery OR minimal clinically important difference OR MCID OR patient-acceptable symptom state OR PASS OR minimally detectable change OR MDC OR clinically important difference OR CID OR substantial clinical benefit OR SCB)) AND ("Patient Outcome Assessment"[Mesh] OR "Patient Reported Outcome Measures"[Mesh] OR "Visual Analog Scale"[Mesh] OR "Knee Injuries"[Mesh] OR "Quality of Life"[Mesh] OR "Lysholm Knee Score"[Mesh] OR PROMS OR Knee Injury and Osteoarthritis Outcome Score OR KOOS OR KOOS activity of day living OR KOOS ADL OR KOOS pain OR KOOS quality of life OR KOOS QoL OR Lysholm knee scoring scale OR Short Form 12 OR SF-12 OR Short Form 36 OR SF-36 OR Cincinnati Knee Rating System OR CKRS OR Western Ontario and McMaster Universities Osteoarthritis OR WOMAC)
