# Supplementary material for: Clinical relevance of patient-reported outcome measures in the surgical management of focal chondral defects of the knee: a systematic review
Source: J Orthop Traumatol. 2026 Jan 16;27:3. doi: 10.1186/s10195-025-00897-0 (PMC12847531; doi:10.1186/s10195-025-00897-0)
Supplement: Supplementary file 2 — Supplementary Material 2. [file 10195_2025_897_MOESM2_ESM.docx]

**Point-to-point Response**

**'Minimal clinically important difference, patient-acceptable symptom state, minimally detectable change, clinically important difference, and substantial clinical benefit in patients following surgical management of focal chondral defects of the knee: a systematic review' (JOOT-D-24-00287)**

**Reviewer #3:**

Dear authors, thank you for considering JOOT for publication. A systematic review was performed to analyse the clinically relevant difference of PROMs after surgical management of focal chondral knee lesions. Out of 524 articles only 4 could be included, which is rather a small number. The study is interesting and valuable because a statistical difference does not automatically mean a clinical difference. However, I am wonder whether the clinical difference of PROMs depends on the pathology or does it rather reflex the subjective outcome of the patients.

The manuscript needs to be revised by a native English-speaking person.

**ANSWER:** Thank you for this comment. The manuscript has been revised by a native academic English-speaker

Specific comments:

TITLE: The title is unclear because you did not define the clinically relevant outcome thresholds rather than the clinical relevance of the PROMs in term of analysing the improvement after surgery.

**ANSWER:** We thank the reviewer for all the time invested. We have revised the title accordingly

ABSTRACT:
Line 30-38: According to the conclusion you mention MCID, CID and MDC only. What about the other parameter? What about the type of cartilage repair? This has been completely neglected.
**ANSWER:** We thank the reviewer for this comment. We have revised the conclusion. Concerning the type of cartilage repair, they were heterogeneous. Given the limited number of studies available, a further subspecification was not possible to conduct. We have now discussed this in the section regarding study limitations.

Line 38: I agree on the last sentence of the conclusion, but what showed the IKDC, which is inappropriate for assessing cartilage repair.

**ANSWER:** We appreciate this aspect raised by the reviewer. We further analysed this point in the section discussed. We agree with the reviewer that the IKDC may not be the optimal PROM for evaluating outcomes after cartilage surgery. This article presents a meta-analysis of the available literature. Because the IKDC was used in one study to assess outcomes after cartilage surgery, we are ethically obligated to report the data. We do, however, state the following in the Discussion: “*More detailed and condition-specific questionnaires might need to be implemented in this context to allow for substantial improvements at a more demanding level, as do these presently established PROMs, such as the KOOS or the IKDC.”*

INTRODUCTION:
It remains unclear why you have performed the analysis specifically for cartilage defects. You should also state why all different calculations were compared, such as MDIC, MDC, CID…..?
**ANSWER:** Thank you for this comment. The current literature is rich in research on the surgical management of knee chondral defects; however, there is a paucity of psychometric scales to assess whether improvements are clinically relevant. Therefore, this study is important for researchers, as it provides a reference to translate their results into a clinically meaningful modality. On the other hand, another strength of the present study is that it conveys a clear message that additional research is needed in this field. We have now inserted the following additional sentence in the introduction:*“To interpret scientific findings correctly, results ought to be placed into a context of such parameters. This systematic review evaluated the* *presently available* *reported data on MCID, SCB, CID, PASS, and MDC of the most commonly applied PROMs used to assess patient outcome following surgical management of focal chondral defects of the knee.”*

Line 43- 47: Please discard. The paragraph is out of context and does not contribute to the purpose of the paper.

**ANSWER:** Thank you for this comment. We have deleted now this paragraph.

Line 52: Please revise the sentence. You may say: "The clinical outcome differs between the size of the defect and repair technique".

**ANSWER:** Thank you for this comment. We have revised the sentence accordingly.

Line 58: Please revise the sentence. I do not understand.

**ANSWER:** We apologise for the inconvenience and have revised the sentence to improve accessibility. The text now runs as follows: *“The exact level of evidence of these recommendations is, however, not always clear. One of the reasons for this is that the reporting of study results often permits considerable misinterpretation.”*

Line 92: Please clear the difference between MDC, MCID and the other analytic methods.
**ANSWER:** Thank you for this comment. Due to the number of parameters included, we tried to keep it as short and concise as possible. Apparently, our wording still left some room for improvement. We have expanded this section to more clearly illustrate the differences between these terms.

You need to explain in the introduction the reason why the analysis of PROMS specifically after cartilage surgery has been performed. Why does the clinical relevant difference of PROMS may differ after ACL reconstruction, Meniscus or cartilage repair?

**ANSWER:** Thank you for this comment. We have discussed this issue in the limitations section.

Line 90-95: Please move to the method section. This is the most important information to understand the study. However, why did you analyse the other parameters. What is the additional information?

**ANSWER:** Thank you for this comment. We have expanded this section according to the reviewer’s suggestion. We also developed a dedicated section on this in the discussion.

METHODS:
Line 118: You did only include arthroscopic procedures? Why did you exclude the open once, which comprise a significant number. However, in table 1 the study by Ogura followed patients after arthrotomy. Please clarify.

**ANSWER:** Thank you for this comment. We included all surgeries addressing chondral defects of the knee. We now make it clearer in the methods section, and we specifically report on this in the results section.

Line 135: Please explain why the different data item were collected and clarify the similarity of difference.

**ANSWER:** Thank you for this comment. The present study systematically reviews the current evidence on the MCID, SCB, CID, PASS, and MDC for the most commonly used PROMs in assessing patients after surgical repair of focal chondral defects of the knee. As suggested by the reviewer, we have expanded the section that explains and defines the different parameters.

Line 139: Why did you choose these PROMs? Not all of them are commonly used for assessment of cartilage procedures.

**ANSWER:** Thank you for this comment. As stated in the methods, all clinical studies investigating tools to assess the clinical relevance of PROMs in the surgical management of focal chondral defects of the knee were accessed. These PROMs were identified through the literature search and are most commonly used to evaluate chondral defects. We agree with the reviewer in that these parameters are not in frequent use yet, and we advocate for changing that.

RESULTS:
Line 173: What do you mean with inadequate studies?

**ANSWER:** Thank you for this comment. We referred to reviews, opinions, letters, and editorials. We specified this now in the running text, and we also replaced the expression “inadequate study design” with the term “type of article”.

Figure 2: According to figure 2 different procedure were included such as OCA, ACI and microfracture. One may expect different outcome, however the difference between prior and after surgery is important and as stated above irrelevant for the procedure.
**ANSWER:** Thank you for this comment which we completely agree with. We now address this aspect in greater detail in the discussion and the study limitations. Due to the paucity of data, we had to include multiple techniques to at least yield meaningful results. We hope that this situation can be resolved in the future.

RESULTS:
Appropriate
**ANSWER:** Thank you for the positive feedback

DISCUSSION:
Start the discussion with the main finding.

**ANSWER:** We appreciate the reviewer's comment. We have now deleted the introductory sentence to shorten the main finding summary. It now comprises only four short sentences: the first relates to the dimensions analysed, the second describes the main findings from the literature search, and the third and fourth report the main results from the meta-analysis of the reported data.

Line 247-250: The sentences are very difficult to follow. You mention different score such as IKDC and KOOS in relation to PASS. Why there are different tools needed for calculation in the clinical relevant difference.

**ANSWER:** Thank you for this comment, which touches upon a highly relevant aspect and problem in the calculation of these values. We have taken this opportunity to further examine this aspect. At the end of the discussion, an entire paragraph is now dedicated to that topic.

Line 251: You mention the IKDC, the most in-appropriate one for assessing the outcome after cartilage surgery. Please comment

**ANSWER:** We agree with the reviewer that the IKDC may not be the optimal PROM for evaluating outcomes after cartilage surgery. This article presents a meta-analysis of the available literature. Because the IKDC was used in one study to assess outcomes after cartilage surgery, we are ethically obligated to report the data. We do, however, state the following in the Discussion: “*More detailed and condition-specific questionnaires might need to be implemented in this context to allow for substantial improvements at a more demanding level, as do these presently established PROMs, such as the KOOS or the IKDC.”*

Line 253: Why these differences occur? Collins et al. reported about knee injuries and not solely cartilage defects, which may the reason for differences. Please discuss.

**ANSWER:** We thank the reviewer for spelling out that point. We have added this aspect not to the manuscript. For further embedding of the topic, please see also our reply and paragraph related to the previous suggestion.

Line 296-301: The paragraph is rather a repetition what has already been discussed.

**ANSWER:** We agree with the reviewer on that aspect. We had introduced this section only in the previous revision of the manuscript at the explicit suggestion of one reviewer. We have now tried to shorten this section to minimise redundancies.

Line 303: Does it make any difference for the analysis whether the cited studies were prospectively or retrospectively designed.

**ANSWER:** We appreciate this very valid comment from the reviewer. We believe it does, however, make a relevant difference, as internal anchor-based data might not always have the necessary discriminatory power in a retrospective dataset as it would if prospectively reported by each patient. We have now added this aspect to the manuscript.

REFERENCES:
appropriate
**ANSWER:** Thank you for your positive feedback

Even though the authors have already revised their manuscript they should try to improve their message. Currently the manuscript is very difficult to read and to understand.
The topic is of great interest because numerous clinical researcher look at the statistical difference but not at the clinical relevant difference.

**ANSWER:** Thank you for your suggestion. We have addressed this point at the end of the discussion, and we hope that the message of the paper is now clearer.

**Reviewer #4:**

Dear Authors, First and foremost, I would like to commend you on the revision of your manuscript and the important work you have undertaken. The topic you address, defining clinically meaningful thresholds in the context of surgical treatment for focal chondral defects of the knee, is both timely and critically relevant in current clinical decision-making. The systematic approach and the clarity gained in the revised version reflect a strong effort to enhance the manuscript's impact and accessibility. While your revision has undoubtedly improved the manuscript, I would like to respectfully point out a few remaining areas that may benefit from further attention:

Lack of critical interpretation of clinical implications: The manuscript presents threshold values (e.g., MCID, PASS) but does not sufficiently explore their practical impact on clinical decision-making. How these thresholds might guide treatment selection or patient counseling in daily orthopedic practice remains unclear.

**ANSWER:** We thank the reviewer for helping us bring our article to a high standard. We acknowledge this aspect raised by the reviewer. Due to the lack of data, we are still cautious about giving specific recommendations. We have, however, expanded the manuscript accordingly.

Limited discussion of study limitations: Although you rightly highlight that only four studies met inclusion criteria, the discussion does not adequately explore the implications of this limitation. A deeper reflection on the potential bias, heterogeneity, and limited generalizability of these findings would add depth and transparency to your conclusions.

**ANSWER:** Thank you for this comment. We have now improved and expanded the limitations accordingly.

Basic statistical analysis: The analyses rely mainly on arithmetic means without providing variability measures such as standard deviations, confidence intervals, or sensitivity analyses. While descriptive, this may underrepresent the methodological robustness expected from a systematic review.

**ANSWER:** We thank the reviewer for highlighting this. We appreciate the concern raised; however, we respectfully point out that the amount of available quantitative data for analyses, e.g., of dispersion indices (SD, IQR, etc.), was highly limited. Indeed, some items contained only one value. We therefore refrained from performing such calculations. We discussed this limitation in the dedicated section at the end of the discussion.

Lack of forward-looking recommendations: The call for more specific PROMs is appreciated but remains generic. Adding suggestions about the characteristics or validation approaches for future condition-specific PROMs would significantly strengthen the value and applicability of your conclusions.

**ANSWER:** We thank the reviewer for pointing this out. We have improved the manuscript accordingly.

No use of visual synthesis (e.g., forest plot): Given that several threshold values were reported across multiple PROMs and studies, the inclusion of a forest plot, even without a formal meta-analysis, would enhance the interpretability of the findings. It would visually support the consistency or variability of reported MCID, PASS, SCB, and other thresholds, and add to the scientific rigour of the review.

**ANSWER:** Thank you for this suggestion. Although a visual synthesis, such as a forest plot, may enhance the readability of systematic reviews, it was not feasible in the present analysis. The main limitation arises from the intrinsic heterogeneity of the available data. Across the included studies, several PROMs were evaluated, each reporting distinct psychometric thresholds, including MCID, MDC, CID, PASS, and SCB. These metrics were derived using variable statistical and anchor-based approaches, often without a uniform definition of improvement or a consistent reference standard. Furthermore, many thresholds were extracted from a single study rather than multiple comparable datasets, resulting in the absence of dispersion measures such as standard deviations or confidence intervals. Under these conditions, constructing a forest plot would provide a misleading visual representation, as it would imply a quantitative synthesis that the underlying data do not support. Therefore, the decision to present the findings in a descriptive, tabulated format was deliberate, ensuring transparency while respecting the methodological constraints imposed by the basis of evidence.

**Reviewer #5:**

Overall opinion. Timely and clinically relevant topic. English quality ok.

Abstract
l.10-12: Use "PROMs" consistently (not "PROMS").

**ANSWER:** Changes made as suggested by the reviewer.

Introduction
l.43: Rephrase "pain- and almost frictionless movement" in "to allow nearly frictionless, pain-free joint motion."

**ANSWER:** Thank you for this comment. However, in accordance with Reviewer 1's recommendation, we have removed this section entirely.

l.97-100: Use past tense consistently ("This systematic review evaluated…").

**ANSWER:** We thank the reviewer for highlighting this. We have now corrected this typo.

Methods
l.116-121 (PICOD): Change "Intervention: Arthroscopic reconstruction" in "Surgical management (arthroscopy or arthrotomy) of focal chondral defects."

**ANSWER:** Thank you for this comment. We have now changed the PICO to be more appropriate for this systematic review.

Results
l.185 Fix typos: "risk of bias."

**ANSWER:** Correction performed as suggested by the reviewer.

Table 1: Correct "Lysohlm" in "Lysholm."

**ANSWER:** We have now corrected this typo, thank you.

Table 2: Clarify n: several subscale n's exceed total patients. State whether n = observations/timepoints or correct the counts.

**ANSWER:** Thank you for this comment. N represents the number of patients included in a determined analysis or paper. We have now clarified this in the text.

Correct "MCS: metal component" → "MCS: mental component summary."

**ANSWER:** Thank you for this comment. We have now corrected this typo.

Verify whether reported SF-36 values are MCID vs MDC (values like 0.3 on a 0-100 scale seem implausible).

**ANSWER:** Thank you for this comment. We have now revised this.

Discussion
l.253-261: When comparing to the literature, indicate whether thresholds are anchor-based or distribution-based.

**ANSWER:** Specified as suggested by the reviewer.

l.305-309: Temper comparisons between CID vs MCID/MDC given heterogeneity and limited data; prefer a descriptive take-home message and call for standardized prospective studies.

**ANSWER:** We thank the reviewer for this comment and absolutely agree. Apparently, our wording was still too brisk. The paragraph now runs as follows: *“The scarceness and heterogeneity of the data also led to seemingly implausible results since values reported for the CID were, for example, mostly smaller than for the MCID and the MDC. We, therefore, strongly advocate a regular use and reporting of such parameters in future studies to allow a better comparability between different studies and treatment results in the future.”*

We hope that the changes implemented have improved the manuscript and that it has now reached the standard necessary for formal acceptance in the *Journal of Orthopaedics and Traumatology*.

We look forward to hearing from you.

Yours sincerely

The Authors
